# Influence of hyperprolactinemia on collagen fibers in the lacrimal gland of female mice

**DOI:** 10.6061/clinics/2015(09)07

**Published:** 2015-09

**Authors:** Ariadne Stavare Leal Araujo, Manuel de Jesus Simões, Carina Verna, Ricardo Santos Simões, José Maria Soares Júnior, Edmund Chada Baracat, Regina Célia Teixeira Gomes

**Affiliations:** I Universidade Federal de São Paulo (UNIFESP), Ginecologia, São Paulo/, SP, Brazil; II Universidade Federal de São Paulo (UNIFESP), Morfologia e Genética,; III Faculdade de Medicina da Universidade de São Paulo, Obstetricia e Ginecologia, São Paulo/, SP, Brazil; IV Universidade Federal de São Paulo (UNIFESP), Oftalmologia, São Paulo/, SP, Brazil

**Keywords:** Hyperprolactinemia, Collagen Fibers, Sex Hormones

## Abstract

**OBJECTIVE:**

To quantify the collagen fibers in the lacrimal gland of female mice with hyperprolactinemia.

**METHODS:**

Forty adult female mice were randomly divided into two groups with 20 animals each: nonpregnant control (CTR1, control group, 0.2 mL of saline solution) and nonpregnant experimental (HPRL1, experimental group, 200 µg/day metoclopramide). Treatments lasted for 50 consecutive days. On day 50, 10 females from each group (control and experimental) were euthanized in the proestrus phase; then, the blood was collected and the lacrimal glands were removed. Thereafter, the remaining females were placed with the mates and continued to receive treatment with saline solution or metoclopramide. On the 6th post-coital day, 10 pregnant females from the control group (CTR2) and 10 pregnant females from the experimental group (HPRL2) were euthanized, after which blood was collected and the lacrimal glands removed. The lacrimal glands were processed for morphological analyses and collagen quantification, and prolactin and sex steroid levels were measured in the blood samples. Data were statistically analyzed using an unpaired Student t test (*p*<0.05).

**RESULTS:**

Morphological analysis revealed greater structural tissue disorganization of the lacrimal glands in the metoclopramide-treated groups. The total collagen content was significantly higher in the HPRL1 group than in the CTR1 group (*p*<0.05), whereas the difference between the CTR2 and HPRL2 groups was not significant.

**CONCLUSION:**

Our data suggest an impairment in the functioning of the lacrimal gland as a consequence of increased prolactin levels and decreased serum levels of estrogen and progesterone.

## INTRODUCTION

Hyperprolactinemia is a constant concern in many areas of medicine, including ocular health, and thus constitutes a multidisciplinary interest. Researchers have reported that prolactin can act on various body systems, in particular the ocular system, thereby influencing the function of the lacrimal gland 1-2. In addition, hormones within the hypothalamic-pituitary-gonadal axis exert a profound impact on the lacrimal gland 3. For example, hypophysectomy or ablation of the pituitary gland induces atrophy tear 4. Prolactin, along with estrogen, progesterone and androgen, is directly related to tissue proliferation and differentiation. Receptors for these hormones have been identified in various ocular tissues of rodents, rabbits and humans 3. In humans and rodents, hyperprolactinemia may negatively affect ovarian function due to elevated prolactin levels, which are associated with the decreased synthesis of sex hormones (estrogen and progesterone) 5-9. The quality of the tear film depends on fine adjustments between neuronal and hormonal mechanisms. A thorough comprehension of the endocrine changes in the lacrimal glands is essential for understanding the effects of hyperprolactinemia. However, there are several questions that remain unanswered; metoclopramide-induced hyperprolactinemia in female mice has been used as a model in investigations of these questions 5-9. Hyperprolactinemia may be related to the etiology of dry eye 10. Dry eye is a complex condition that involves multiple factors. In the last decade, research in animals and humans showed that dry eye syndrome is usually a result of an inflammatory condition caused by an imbalance in sex hormones 11. These studies suggested that specific hormonal levels are necessary for maintaining the anti-inflammatory and secretory functions of the entire ocular surface system, including the lacrimal and meibomian glands (tarsal) 12. It should be noted that patients with Sjögren's syndrome have eye dryness and high serum levels of prolactin 10, but most cases occur without the typical signs of Sjögren's syndrome. They occur more frequently in women than in men and are often associated with altered states of the systemic hormonal milieu: perimenopause, postmenopause, pregnancy, lactation, oral contraceptive use, and estrogen or estrogen-progesterone replacement therapy. Among men, the frequency increases with age 13. However, the relationship between hyperprolactinemia and dry eye syndrome is not well defined. Furthermore, the physiological role of prolactin in the lacrimal glands is not completely understood. Lacrimal gland failure due to inflammation is a common condition that results in the symptoms of dry eye, which affects an estimated 1–4 million North Americans (mostly women) 14-15. The current worldwide prevalence of dry eye is estimated at approximately 11% to 22% 16. Much of the basic architecture and functional properties of Sjögren's syndrome are dictated by connective tissue. Connective tissue components, such as collagen, provide the substrate that supports the lacrimal gland acinar cells, providing the signal that triggers selective development and differentiation in response to pressure, which leads to the loss of cells due to necrosis or apoptosis. In fact, changes in connective tissue have been implicated as possible contributors to alterations in glandular function. It is important to note that changes in the collagen fiber content can interfere with the function of the lacrimal gland because these fibers they are involved in structural changes, and depending on the extent of these changes, fibrosis can occur 14. Collagen fibers are the most abundant protein in the extracellular matrix (ECM). Collagen is present in the ECM as a fibrillar protein, where it provides structural support to resident cells and participates in cell-cell and cell-matrix interactions. Moreover, collagen has a fundamental role in tissue architecture 17. Verna et al. (2005) 5 showed that metoclopramide-induced hyperprolactinemia produced morphological signs of reduced cellular activity in the lacrimal glands during the proestrus phase and pregnancy, but several questions remain unanswered. The specific objective of this study was to evaluate the amount of collagen in the lacrimal gland of female nonpregnant and pregnant mice with metoclopramide-induced hyperprolactinemia.

## MATERIALS AND METHODS

The experiments were performed at the Department of Histology of the Federal University of São Paulo – Escola Paulista de Medicina (UNIFESP-EPM). The protocols were designed according to the Committee of Ethics on Animal Experimentation (Report No. 0333/10) and approved by the Institutional Review Board of UNIFESP-EPM. A total of 40 virgin adult (3-month-old) female mice (Mus musculus) and 5 fertile male mice, all weighing 35-40 g, were given food and water ad libitum at room temperature (22 °C) under artificial light with a 12:12 h light:dark photoperiod (lights on from 7 a.m.–7 p.m.). The animals were undisturbed in the new environment for 2 weeks. Thereafter, vaginal smears were taken daily to evaluate the stage of the estrous cycle. The animals were considered to be cycling when they exhibited 4- to 5-day regular consecutive estrous cycles. Then, 40 animals were randomly divided into two groups with 20 animals each: the control group (CTR1) received subcutaneous injections of 0.2 mL of saline solution for 50 consecutive days, and the experimental group (hyperprolactinemic, HPRL1) received subcutaneous injections of 200 µg/day metoclopramide dissolved in 0.2 mL of saline solution for 50 consecutive days 7. The animals were always treated at 12:00 a.m. On the 50th day, 1 h after the last injection, colpocytological examinations of the vaginal smears were performed, and 10 females in the control group and 10 females in the experimental group (proestrus phase) were euthanized by guillotine decapitation; then, blood was collected for hormone measurements, the lacrimal glands were collected, and both sets of samples were evaluated according to the protocol described below. These groups were termed the nonpregnant control (CTR1) and nonpregnant experimental (HPRL1) groups. Then, the remaining females (10 females in the control group and 10 females in the experimental group, all in estrus) were placed with the mates, with three females for each fertile male. Mating was demonstrated by the presence of a vaginal plug or the presence of spermatozoa in the histological slide. Then, the females in the control and experimental groups continued to receive treatment with saline solution or metoclopramide, respectively. On the 6th post-coital day, the pregnant females were euthanized by guillotine; then, blood was collected for hormone measurements, the lacrimal glands were collected, and both sets of samples were evaluated according to the protocol described below. These groups were termed the pregnant control (CTR2) and pregnant experimental (HPRL2) groups.

### Protocol

Blood serum samples were separated by centrifugation for 10 minutes at 3,000 rpm and stored at -20 °C for subsequent hormone measurements. The lacrimal glands were removed and fixed for 24 h in 10% formaldehyde in phosphate-buffered saline (PBS).

### Histological processing

After fixation, tissue samples were processed for paraffin inclusion by dehydration in ethanol, diaphonization in xylol, and impregnation in liquid paraffin in a drying oven at 60 °C. The 3-μm sections of the paraffin-embedded blocks were cut using a Minot microtome (Leika®, Germany). Three of the sections were stained using Masson's trichrome method 18 to quantify collagen (blue) in the connective tissue within the lacrimal gland.

### Photomicrographs

The photomicrographs were collected using a computerized system consisting of a light microscope (Axiolab Standard 2.0, Carl Zeiss, Germany) connected to a high-resolution camera (300 dpi) (AxioCam, Carl Zeiss, Germany) and a color video monitor (Samsung) at a magnification of 100× to 400×.

### Quantification of collagen

**Figure 1:**
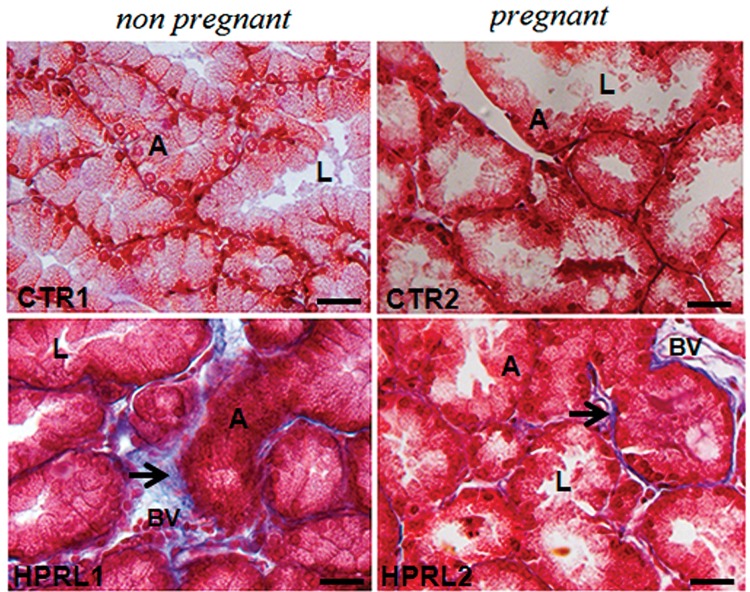
Photomicrographs of lacrimal glands in the nonpregnant groups (CTR1 and HPRL1) and the pregnant groups (CTR2 and HPRL2) after Masson's trichrome staining. Note the blue collagen fibers (arrow), the lumen (L) and the acinar cells (A). The bar corresponds to 400x magnification.

**Figure 2:**
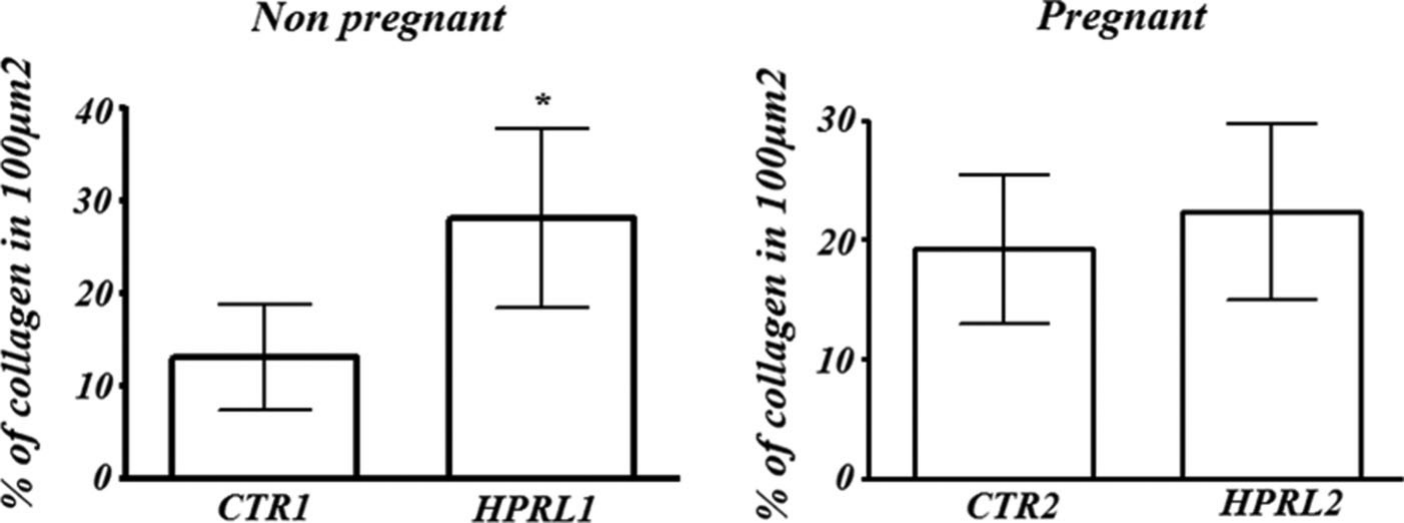
Quantification of collagen fibers in the lacrimal gland in nonpregnant and pregnant female mice with hyperprolactinemia induced by metoclopramide.

The photomicrographs were used for the semiquantification of collagen fibers (blue) in the lacrimal glands using ImageLab® processing and image analysis software (Computer Software Ltd, Brazil). For each lacrimal gland, 10 histological fields per section were evaluated to cover the entire length of the cut, enabling a complete analysis. The analyses were performed by two independent researchers who were blinded to the sample identity. The calculations were repeated for each animal. The results are expressed as % collagen per μm^2^ (Figures 1 and 2).

### Hormone measurements

Serum estradiol and progesterone concentrations were measured using a radioimmunoassay (RIA) kit according to a previously described method using the appropriate kits for estradiol (Coat-a-Count TKE21, Siemens, CA, USA) and progesterone (Coat-a-Count TKPGI, Siemens, CA, USA). The samples were assayed in triplicate. The sensitivities of the assays were 8 pg/mL for estradiol and 0.02 ng/mL for progesterone. Quantifications were performed with a Wizard model 1470 reader (Perkin-Elmer-Wallac, Inc. Turku, Finland). The prolactin concentration was measured using an enzyme-linked immunosorbent assay (ELISA) for mouse prolactin (E90846Mu enzyme-linked immunosorbent assay kit, USCNK, Houston, TX, USA). Quantifications were performed with a Stat Fax® 2100 photometer system (Fisher Bioblock Scientific, USA), and the data were analyzed with MultiCalc® software (Perkin-Elmer-Wallac, Inc., Turku, Finland). The prolactin assay sensitivity was 0.055 ng/mL. No significant cross-reactivity or interference between mouse PRL and its analogues was observed. The testosterone cross-reactivity was 0.1% for the progesterone kit and 0.001% for the estradiol kit. Quantifications were performed by the Genese Institute of Scientific Analysis, São Paulo, Brazil (Table 1).

### Statistical analysis

The measurements were subjected to a normality test (Kolmogorov-Smirnov test). The statistical analyses of the nonpregnant and pregnant groups were conducted separately because the physiological state of pregnancy evokes particular hormonal profiles. The unpaired Student t test was used to compare the nonpregnant groups (CTR1 versus HPRL1) and the pregnant groups (CTR2 versus HPRL2). The data are presented as the mean ± standard deviation (SD). All statistical tests were performed using GraphPad Prism software version 3.0 for Windows (GraphPad Software, San Diego, CA, USA). The significance level was set at *p*≤0.05 for all statistical tests.

## RESULTS

### Morphologic analysis

In all the analyzed groups, the lacrimal glands showed no change in typical architecture: the lacrimal gland appeared as a capsule of connective tissue (collagen fibers in blue) that divided the gland into lobules. The basal cells (myoepithelial) and cylindrical cells (acinar cells) surrounded the central lumen with globular structures, similar to secretory vesicles, in the cytoplasm.

However, morphological changes were observed in the nonpregnant experimental group (HPRL1) compared to the nonpregnant control group (CTR1): cellular disorganization was observed, and some acinar cells had a cylindrical shape, whereas others had a cubic shape or a rounded morphology. In some areas, it was not possible to visualize the lumen and the cell boundaries. There were more collagen fibers (blue) in the connective tissue around each lobe and on the capsule in the HPRL1 group than in the CTR1 group. Interestingly, the morphology of the lacrimal glands in the nonpregnant group was similar to that of those in the pregnant control group (CTR2) (Figure 1).

The pregnant control group (CTR2) and the experimental pregnant group (HPRL2) had similar lacrimal gland morphology: most of the cells had a cubic shape. Round structures were visible on the apical surface (secretory vesicles). Collagen fibers (blue) were also observed in the connective tissue around each lobe and on the capsule in the HPRL2 and CTR2 groups. The greatest morphological difference was that the lacrimal gland exhibited larger acini and a broader lumen in the pregnant experimental group (HPRL2) compared to the pregnant control group (CTR2) (Figure 1).

### Quantification of collagen

The results of the % of collagen fibers in the lacrimal glands are summarized in Figure 2.

**In the nonpregnant groups:** The % of collagen fibers in the lacrimal gland was higher in the nonpregnant experimental group (HPRL1) compared to the nonpregnant control group (CTR1) (*p*<0.05).

**In the pregnant groups:** There was no significant difference between the HPRL2 and CTR2 groups.

### Hormone measurements

The results of the ovarian steroid and prolactin measurements are summarized in Table 1.

**In the nonpregnant groups:** The serum prolactin levels were elevated in the nonpregnant experimental group (HPRL1) compared to the nonpregnant control group (CTR1) (*p*<0.05).

**In the pregnant groups:** The estrogen and progesterone serum levels were lower in the HPRL1 group compared to the CTR1 group (*p*<0.05).

Serum prolactin levels were higher in the pregnant experimental group (HPRL2) compared to the pregnant control group (CTR2) (*p*<0.05).

The estrogen and progesterone serum levels showed no significant difference between the HPRL2 and CTR2 groups.

## DISCUSSION

Initially, the state of hyperprolactinemia was correlated with infertility in humans based on the negative effects on ovarian function, leading to changes in the serum levels of sex hormones 6-7. Prolactin acts systemically through its receptor in target organs, such as the uterus, cornea and lacrimal gland 1,12-16,. In the present study, the serum prolactin levels were elevated in all the metoclopramide-treated animals. The serum levels of progesterone and estrogen were lower in the nonpregnant experimental females than in the nonpregnant control females. In addition, in pregnant mice treated with metoclopramide, the estrogen and progesterone serum levels were not significantly different from those in the control pregnant mice. These results suggest a compensatory mechanism in pregnant animals. However, there are several unanswered questions regarding the influence of elevated prolactin levels on the lacrimal glands. The main condition related to a sex hormone (estrogen/androgen) imbalance and prolactin is Sjögren's syndrome, also known as dry eye syndrome, which is also associated with high levels of proinflammatory cytokines in the tear film and ocular surface epithelium 20-21. In humans and mice, the lacrimal gland is composed of three major types of cells: acinar, ductal and myoepithelial cells. In addition, plasma cells, lymphocytes, macrophages, and mast cells are present. Acinar cells are responsible for producing the tear film, which is very important for lubricating, protecting, and nourishing the eyes as well as providing a barrier against foreign infectious agents. The stroma is composed of connective tissue, blood vessels and nerve fibers. The connective tissue is formed by extracellular matrix, which contains collagen fibers and amorphous ground substance. Collagen is the major component of connective tissue and plays a crucial role in tissue architecture 22-24. Our study revealed morphological changes in the lacrimal glands of female mice with hyperprolactinemia induced by metoclopramide; these changes included cellular disorganization, changes in the volume of acinar cells and altered spacing between the acini that form the lacrimal gland. Verna et al. (2005) 5 analyzed the morphology of the lacrimal glands using the same experimental model and associated these changes with signs of reduced cellular activity in lacrimal glands during proestrus and pregnancy. However, several questions remained unanswered. Moreover, in the present study, the major morphological changes in the metoclopramide-treated animals were the size of the acinar cells, irregularities in the cell boundaries and greater spacing between the acini of the lacrimal gland. In the nonpregnant animals treated with metoclopramide, there were more cylindrical and/or cubic acinar cells with rounded morphology, more irregular cell boundaries, more space between the acini that comprise the gland, and a narrower acini lumen compared to the control nonpregnant animals. In contrast, in the pregnant animals treated with metoclopramide, there were more cubic acinar cells with round structures on their apical surface and a broader acini lumen compared to in the control pregnant animals. Our results showed an increase in the amount of collagen fibers in the nonpregnant and pregnant animals treated with metoclopramide. Nevertheless, these changes were greater in animals with nonpregnant hyperprolactinemia (proestrus phase) compared to control animals. Moreover, the connective tissue in the human and mouse lacrimal glands is a complex network formed by collagen, elastic fibers and reticular fibers (collagen type III) capable of forming an environment that supports migration, development and extracellular matrix-cell interactions [17-13]. In a process of constant aggression, inflammation or delays in cell division can cause fibrogenesis as a result of an imbalance between the synthesis and degradation of extracellular matrix components. Clinically, this condition would lead to hardening of the lacrimal gland, thus damaging its function. Such conditions are also present in dry eye syndrome 14. Histopathological studies of normal subjects showed that with advancing age, processes such as periductal fibrosis, interacinar fibrosis and ultimately acinar atrophy are associated with the primary deficiency of the lacrimal gland, leading to dry eye 14. In recent decades, several studies in animals and humans have been conducted to elucidate the relationship between hormonal changes and the ocular system. Despite the large number of studies to date, several questions remain unanswered and require further investigation. In the present study, the irregularities observed in the cell boundaries may represent a disconnection among these cells because they are held together by cell junctions that provide cell-cell and cell-ECM contacts. Our observations were limited by the utilized methodology; further studies using electron microscopy can confirm these findings. The presence of tight junctions results in the formation of highly polarized cells and the division of the plasma membrane into the apical and basal membranes. These events ensure the unidirectional secretion of secretory products toward the ductal lumen. Receptors for hormones and neurotransmitters are located on the basolateral membrane. The secretion of electrolytes and water into the lumen is dependent on the activation of ion transport proteins and ion channels located on the apical and basolateral membranes 13,25. These results suggest that higher levels of prolactin (hyperprolactinemia) might interfere with signaling pathways via hormone receptors and neurotransmitters, thereby changing the functioning of the lacrimal glands 15,21,.

In summary, our data show alterations in acinar cells and in the amount of collagen in the lacrimal gland in female mice with hyperprolactinemia induced by metoclopramide. These data suggest an impairment in the functioning of the lacrimal gland as a consequence of elevated prolactin levels and decreased serum levels of estrogen and progesterone.

## Figures and Tables

**Table 1:** Hormone measurements in nonpregnant and pregnant female mice with hyperprolactinemia induced by metoclopramide.

Hormone levels	Nonpregnant		Pregnant	
	CTR1	HPRL1	CTR2	HPRL2
Estradiol (pg/mL)	47.2±3.3	39.4±2.1*	84.4±5.6	79.6±6.4
Progesterone (ng/mL)	3.8±0.2	3.4±0.5*	24.5±2.9	26.8±3.6
Prolactin (ng/mL)	37.98±4.2	82.65±5.3*	98.3±15.6	152.12±16.5**

Note: Number of animals=10. Data are presented as the mean ± standard deviation. The *p* values represent the comparisons between the nonpregnant groups (CTR1 versus HPRL1) and the pregnant groups (CTR2 versus HPRL2 groups): unpaired Student's t test, *p*<0.05 = significant. The superscripted * indicates significant differences: ***estradiol levels***: *p<0.05 compared to the CTR1 group (nonpregnant); ***progesterone levels***: * *p*<0.05 compared to the CTR1 group (nonpregnant); ***prolactin levels***: * and ** *p*<0.05 compared to the nonpregnant group (CTR1) and to the pregnant group (CTR2).
